# Supplementary material for: Perspectives from a multi-stakeholder workshop for implementing a risk-stratified breast cancer screening program in Canada
Source: Commun Med (Lond). 2026 Apr 21;6:242. doi: 10.1038/s43856-026-01582-x (PMC13109382; doi:10.1038/s43856-026-01582-x)
Supplement: Supplementary file 2 — Supplemental Information [file 43856_2026_1582_MOESM2_ESM.pdf]

Supplementary Tables

Supplementary Table 1: Breakdown of workshop sessions

Session 1	Session 2	Session 3	Session 4
Each working group:<br>• Created pathway maps for an RSBS programme<br>• Identified necessary changes within existing breast screening programmes<br>• Discussed potential feasibility to implement changes	Participants were presented 8 areas* related to RSBS implementation. After individually ranking them and group discussions, four priority areas were ranked in order (based on feasibility and importance) to focus on during the workshop:<br>1) **Defining Care Pathways**<br>2) **Implementation Roadmap**<br>3) **Stakeholder Engagement**<br>4) **Workforce Education and Training**	Each working group focused on one of the four priority areas to identify challenges and opportunities, developed strategic objectives and an action plan	Each working group developed mitigation plans related to the strategic objectives of their priority area

* Workforce education and training; Stakeholder engagement – motivating and securing commitment; Public engagement – raising awareness; Defining care pathways – invitation, risk assessment and integration; Communications: invitations for risk assessment, risk results, and screening recommendations; Making implementation of RSB a government priority; Equitable delivery of RSBS; Implementation roadmap – leveraging existing infrastructure and stepwise approach
Abbreviations: RSBS, risk-stratified breast screening

Bold text indicates the four priority areas selected and ranked by participants based on perceived feasibility and importance

Supplementary Table 2: Characteristics of workshop participants

**Characteristics**	**N**
Invited participants *	34
**Role(s) †**	
Provincial/territorial government role	14
Breast screening program manager/director	17
Healthcare practitioner (e.g., family physician, radiologist)	8
Patient/public advocate	1
Representatives of patient/public organizations ‡	2
**Province/Territory**	
Quebec	12
Ontario	13
Manitoba	2
Newfoundland and Labrador	2
Prince Edward Island	1
Nova Scotia	1
Alberta	1
Nunavut	1
Saskatchewan	1
**Gender**	
Women	28
Men	6

* In addition to invited participants, there were 8 facilitators

† Roles are not mutually exclusive

‡ Includes Quebec Breast Cancer Foundation (n=1); Rethink Breast Cancer (n=1)

Supplementary Table 3: Stakeholder groups and levels relevant for implementing RSBS in Canada

**Level**	**Examples of stakeholder groups**
**Government and Health Authorities**	Provincial/territorial Ministries of Health<br><br>Federal Agencies<br>(e.g. Health Canada, the Public Health Agency of Canada)<br><br>Canadian Partnership Against Cancer (CPAC)<br><br>Canadian Breast Cancer Screening Network (CBCSN)<br><br>Health informatics leads/Data governance bodies
**Screening programmes**	Breast Cancer Screening Programmes' coordinators<br><br>Provincial/Territorial cancer agencies<br><br>Quality assurance and evaluation teams
**Healthcare Communities**	Healthcare professionals<br>(e.g., family physicians, nurse practitioners, radiologists, medical radiation technologists, genetic counsellors, oncologists and breast health specialists etc.)<br><br>Medical and professional associations<br>(e.g., College of Family Physicians of Canada, Canadian Association of Radiologists, Canadian Society of Breast Imaging, etc.)
**Public, patient and community groups**	General public (as a whole)<br><br>Advocacy organisations<br><br>Breast cancer survivors and lived-experience representatives<br><br>Ethnic minority community representatives<br><br>First Nations, Inuit and Métis communities and/or organisational representatives<br><br>Media<br><br>Community-based organisations promoting screening awareness
**Evidence generators and policy advisors**	Researchers and academic institutions<br><br>Health ethicists and legal experts<br><br>Heath policy organisations/Health Technology Assessments<br>e.g., Institut national d'excellence en santé et en services sociaux (INESSS)

	Canadian Task Force on Preventive Health Care (CTFPHC)
**Funders and support organisations**	Federal and provincial funding bodies<br><br>Non-profit and charitable organisations<br>(e.g., Canadian Cancer Society, Quebec Breast Cancer Foundation)<br><br>Private sector funders supporting research and innovation<br><br>Ministries of Finance / Treasuries
